# Towards Anion Recognition and Precipitation with Water-Soluble 1,2,4-Selenodiazolium Salts: Combined Structural and Theoretical Study

**DOI:** 10.3390/ijms23126372

**Published:** 2022-06-07

**Authors:** Alexey A. Artemjev, Anton P. Novikov, Gleb M. Burkin, Alexander A. Sapronov, Alexey S. Kubasov, Valentine G. Nenajdenko, Victor N. Khrustalev, Alexander V. Borisov, Anatoly A. Kirichuk, Andreii S. Kritchenkov, Rosa M. Gomila, Antonio Frontera, Alexander G. Tskhovrebov

**Affiliations:** 1 Research Institute of Chemistry, Peoples’ Friendship University of Russia, 6 Miklukho-Maklaya Street, 117198 Moscow, Russia; artemyev-ala@rudn.ru (A.A.A.); tony.novickoff@yandex.ru (A.P.N.); burkin-gm@rudn.ru (G.M.B.); aleks.sapronov2013@yandex.ru (A.A.S.); khrustalev-vn@rudn.ru (V.N.K.); kirichuk-aa@rudn.ru (A.A.K.); platinist@mail.ru (A.S.K.); 2 Frumkin Institute of Physical Chemistry and Electrochemistry, Russian Academy of Sciences, 31 Bldg 4, Leninsky Prosp., 119071 Moscow, Russia; 3 Kurnakov Institute of General and Inorganic Chemistry, Russian Academy of Sciences, 31 Leninsky Prosp., 119071 Moscow, Russia; fobosax@mail.ru; 4 Department of Chemistry, M.V. Lomonosov Moscow State University, 1, Leninskie Gory, 119991 Moscow, Russia; 5 N.D. Zelinsky Institute of Organic Chemistry, Russian Academy of Sciences, 47 Leninsky Prosp., 119071 Moscow, Russia; 6 R.E. Alekseev Nizhny Novgorod State Technical University, Minin St., 24, 603155 Nizhny Novgorod, Russia; avb1955@rambler.ru; 7 Departament de Química, Universitat de les Illes Balears, 07122 Palma de Mallorca, Spain; rosa.gomila@uib.es (R.M.G.); toni.frontera@uib.es (A.F.)

**Keywords:** selenodiazoles, non-covalent interactions, chalcogen bonding, anion recognition, rhenium, technetium

## Abstract

The synthesis and structural characterization of a series of supramolecular complexes of bicyclic cationic pyridine-fused 1,2,4-selenodiazoles with various anions is reported. The binding of trifluoroacetate, tetrachloroaurate, tetraphenylborate, perrhenate, and pertechnetate anions in the solid state is regarded. All the anions interact with selenodiazolium cations exclusively via a pair of “chelating” Se⋯O and H⋯O non-covalent interactions, which make them an attractive, novel, non-classical supramolecular recognition unit or a synthon. Trifluoroacetate salts were conveniently generated via novel oxidation reaction of 2,2′-dipyridyl diselenide with bis(trifluoroacetoxy)iodo)benzene in the presence of corresponding nitriles. Isolation and structural characterization of transient 2-pyridylselenyl trifluoroacetate was achieved. X-ray analysis has demonstrated that the latter forms dimers in the solid state featuring very short and strong Se⋯O and Se⋯N ChB contacts. 1,2,4-Selenodiazolium trifluoroacetates or halides show good solubility in water. In contrast, (AuCl_4_)^−^, (ReO_4_)^−^, or (TcO_4_)^−^ derivatives immediately precipitate from aqueous solutions. Structural features of these supramolecular complexes in the solid state are discussed. The nature and energies of the non-covalent interactions in novel assembles were studied by the theoretical methods. To the best of our knowledge, this is the first study that regards perrhenate and pertechnetate as acceptors in ChB interactions. The results presented here will be useful for further developments in anion recognition and precipitation involving cationic 1,2,4-selenodiazoles.

## 1. Introduction

The search for new anion receptors and exploration of novel modes of anion binding is a topic of considerable interest in supramolecular chemistry. Design and synthesis of receptors capable of selective and strong anion recognition has recently emerged as a major challenge [1,2]. Although the area is rapidly developing, an efficacy of natural anion-binding proteins is not yet achieved for artificial systems [3]. This stimulates a significant effort from the synthetic chemistry community in creating novel anion receptors, which can revolutionize several areas, including catalysis, sensing, extraction, and anion transport [4,5,6]. Hydrogen bonding (HB) is usually considered a key interaction, which is employed for anion recognition [7,8]. However, non-classical non-covalent interactions have gained an increase in interest in recent years [9,10,11,12,13,14]. Halogen bonding (XB) has emerged as a powerful alternative to HB, since both interactions have a comparable strength, but XB exhibits a remarkable directionality. In 2014, Beer reported a receptor for anion binding in water, which employed XB and demonstrated a superiority of XB over HB for anion binding in water [1]. Chalcogen bonding (ChB), which also shows a good directionality and tunable strength, is even less explored.

Recently, we discovered a remarkably efficient cyclization reaction between 2-pyridylselenyl halides and nitriles, which allows the synthesis bicyclic cationic pyridine-fused 1,2,4-selenodiazoles [15,16]. Novel heterocycles feature electron-deficient selenium centers, which provide two σ-holes along the extension of the covalent bond axis. Moreover, 1,2,4-selenodiazolium cations bind halide anions via a combination of ChB and HB. The latter supramolecular structural motif was observed for all structurally characterized selenodiazolium halides derived from 2-pyridylselenyl halides and nitriles [15,16,17,18]. However, currently, there is no data about binding modes and energies of 1,2,4-selenodiazoliums with other anions.

The solubility of novel selenium compounds in aqueous media makes them attractive for potential applications, including anion recognition and precipitation, which can be employed in radioactive waste management. For instance, Technetium (^99^Tc) is a by-product of the nuclear fuel cycle with a long half-life (2.1 × 10^5^ years), which is among most problematic elements of utilized nuclear fuel [19]. To date, for the processing of irradiated nuclear fuel, the PUREX method is used, which most often involves the dissolution of fuel in concentrated nitric acid and subsequent sorption of the U and Pu from a solution [20,21]. The long half-life of ^99^Tc and its ability to form anionic particles pose a serious problem for the long-term disposal of radioactive waste. Current methods for removing fission products in reprocessing plants involve cationic species. Thus, there is a strong incentive for a separation of the technetium (usually in the form of the pertechnetate).

Here, we report the synthesis and structural characterization of the 1,2,4-selenodiazolium salts with various anions, exhibiting directional chalcogen bonding interactions that dictate the position of the counterion even in the presence of strong, but non-directional, electrostatic ion-pair interaction. The contributions of chalcogen bonding and hydrogen bonding interactions to the formation of the assemblies in the solid state have been evaluated using the quantum theory of atoms-in-molecules and the electron density parameters.

## 2. Results and Discussion

Earlier, we showed that 2-pyridylselenyl halides (Cl (**2**), Br (**3**)) readily react with nitriles forming cyclic adducts [15,16]. Selenodiazoles, synthesized via a highly efficient newly discovered cyclization reaction, represent novel donors of ChB. The fact that these adducts are soluble in aqueous media makes them attractive objects for the investigation of their anion recognition properties. In our previous works, we described selenodiazolium salts, which carried only chloride or bromide as counterions.

**Scheme 1:**
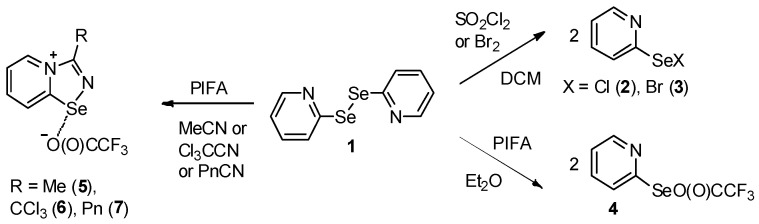
Synthesis of **4**–**7**.

Electrophilic reagents **2** and **3** could be easily generated via oxidation of diselenide **1** by Br_2_ or SO_2_Cl_2_ (Scheme 1). Within this work, we show that the treatment of **1** with bis(trifluoroacetoxy)iodo)benzene (PIFA) in Et_2_O results in the Se–Se bond cleavage and formation of 2-pyridylselenyl trifluoroacetate **4** (Scheme 1). When the reaction was performed in acetonitrile, trichloroacetonitrile, or hexanenitrile, the corresponding adduct **5**–**7** gradually precipitated from the reaction mixtures (Scheme 1), which suggested that in situ generated **4** rapidly reacts with nitriles and its isolation is not necessary. This simple methodology allowed the preparation of selenodiazolium salts **4**–**7,** which contained trifluoroacetate anion.

**Figure 1:**
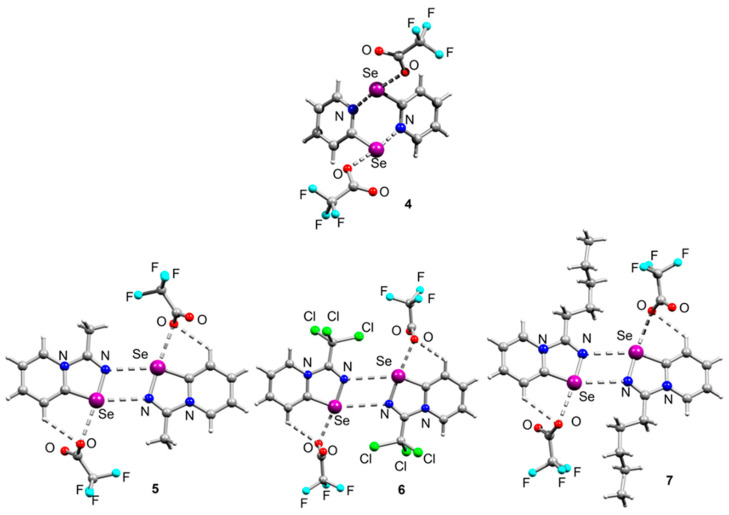
Ball-and-stick representations of the crystal structures of **4**–**7** demonstrating supramolecular dimerization via Se⋯N ChB. Grey and light-grey spheres represent carbon and hydrogen, respectively.

Compounds **4**–**7** precipitate from the reaction mixtures, as well-shaped crystals suitable for the X-ray structural analysis (Figure 1).

2-Pyridylselenyl trifluoroacetate **4** forms supramolecular dimers in the solid state via a pair of equivalent Se⋯N ChB interactions (Figure 1). The Se centers adopt T-shaped geometry (∠N⋯Se⋯O 170.55°). Overall, the bonding situation in **4** is similar to what we observed earlier for PySeCl **2**. In both compounds, the anion occupies a *trans* position against the N atom of the pyridyl in the solid state. A remarkable distinctive feature of **4** is short Se⋯O and Se⋯N distances (2.11 and 2.10 Å), which indicates the significant covalent character of these bonds. To shed light onto this matter, the QTAIM analysis of the dimer has been performed and the degree of covalency has been evaluated by the analysis of the total energy densities and Laplacian of the electron density. That is, in typical closed shell noncovalent interaction, the Laplacian (∇^2^ρ) of the electron density at the bond critical point (CP) that characterizes the contact is positive, whilst it is negative in covalent bonds (∇^2^ρ < 0) [22]. In bonds with partial covalent character (for instance coordination bonds between ligands and metal centers), the Laplacian is positive (∇^2^ρ > 0) and the total energy density (H_r_) at the bond CP is negative (|V_r_| > G_r_), whilst in most weaker noncovalent contacts, such as hydrogen bonds, halogen bonds, chalcogen bonds, etc., both the ∇^2^ρ and H_r_ values are positive. Therefore, the QTAIM parameters are very useful to differentiate covalent, noncovalent, and “partial” covalent bonds. Furthermore, the strength of chalcogen and hydrogen bonds can be derived from the potential energy density (V_r_) using the equations proposed in the literature [23,24] (E ≈ 0.5 × V_r_ for HBs and E ≈ 0.37 × V_r_ − 0.9 for ChBs, V_r_ in kcal/mol).

**Figure 2:**
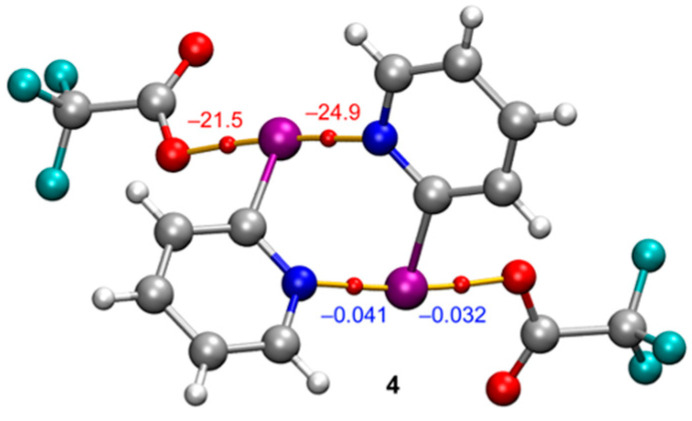
QTAIM analysis of compound **4**. Only intermolecular bond CPs and bond paths are represented. The interaction energies of the ChBs are indicated next to the bond CPs (small red spheres) in kcal/mol. The values of H_r_ (in a.u.) are indicated in blue next to the bond CPs.

The QTAIM analysis of the dimer of compound **4** is shown in Figure 2, where the strength of each ChB is indicated in red next to the bond CPs (represented as red spheres) and the H_r_ values are indicated in blue. In all cases studied herein, the Laplacian values are positive, indicative of closed shell interactions. The ChBs in **4** are characterized by the corresponding bond CPs and bond paths (represented as orange lines) connecting the Se atom to both the O and N atoms of trifluoroacetate and pyridine, respectively. Both ChB contacts are very strong, the latter being slightly stronger than the former. The H_r_ values are in both cases negative, disclosing a partial covalent character, in line with the short distances and strong interaction energies.

The C=N bond lengths (1.27–1.30 Å for **5**–**7**) are typical for C=N double bonds [10,11,13,14,25,26,27,28,29,30,31,32,33,34]. Other covalent bonds in **5**–**7** are unremarkable. The adducts **5**–**7** form supramolecular dimers in the solid state via a pair of equivalent Se···N chalcogen bonds (Figure 1). The formation of similar 2Se–2N squares was observed earlier for the adducts of PySeCl **2** with acetonitrile and trichloroacetonitrile. In contrast, the adduct of **2** with hexanenitrile formed supramolecular polymers via Se⋯Cl and H⋯Cl interactions [18]. Thus, the replacement of chloride by TFA in pentyl-substituted 1,2,4-selenodiazolium salt had a dramatic impact on the self-organization of the compound in the solid state.

Importantly, the TFA anion in **5**–**7** is involved in bifurcated non-covalent interactions (*viz*. Se⋯O and H⋯O, Figure 1), which form a robust chalcogen-bonded supramolecular synthon. So far, we have not observed the anion occupying any other position; it was always found to be involved in “chelating” Se⋯A and H⋯A interactions.

**Figure 3:**
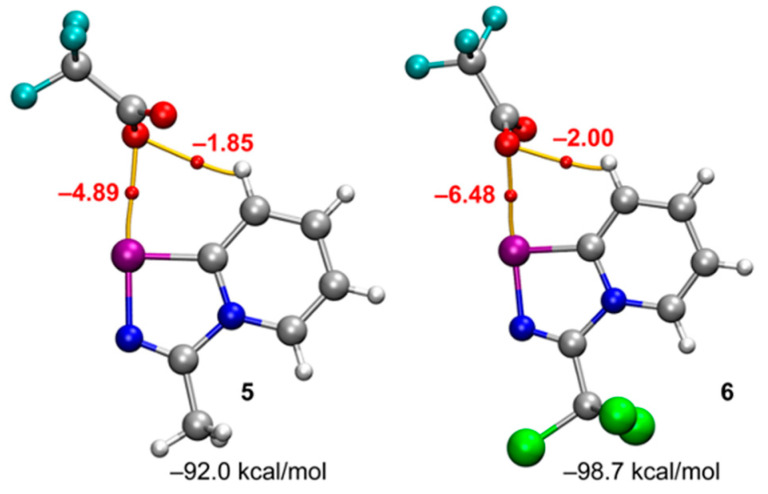
QTAIM analysis of compounds **5** and **6**. Only intermolecular bond CPs and bond paths are represented. The energies of the ChB and HB contacts are indicated next to the bond CPs (small red spheres) in kcal/mol.

It should be noted that the Se⋯O ChB interactions found for **5**–**7** (2.68, 2.56, and 2.68 Å) are unusually short and among the shortest Se⋯O non-covalent interactions involving organoselenium species [35], which is likely due to the cation···anion nature of the interaction. Figure 3 shows the QTAIM analyses of **5** and **6** as representative complexes. In both compounds, the TFA is connected to the 1,2,4-selenodiazolium via two bond CPs and bond paths that characterize the chalcogen and hydrogen bonds. The ChBs are significantly stronger than the HBs (around 2 kcal/mol). Moreover, the strength of the ChB is higher in **6** (−6.48 kcal/mol) than in compound **5** (−4.89 kcal/mol), likely due to the presence of the electron withdrawing the CCl_3_ group in compound **6** instead of the electron donating methyl group in **5**. The dimerization energies are also indicated in Figure 3, which are very large (−92.0 and −98.7 kcal/mol for **5** and **6**, respectively) due to the ion-pair nature and dominance of the Coulombic attraction between counterions.

Further, we were interested how other anions would bind 1,2,4-selenodiazolium cations. For this purpose, 1,2,4-selenodiazolium chloride **6′**, derived from the coupling between trichloroacetonitrile and 2-pyridylselenyl chloride, was chosen for further anion variations. In addition, compound **6′** is very soluble in water, which makes it attractive for anion precipitation purposes. The addition of NaNO_3_ or HBF_4_ to **6′** in water does not result in any precipitation. However, the addition of aqueous NaAuCl_4_, perrhenic, or pertechnetic acids to the aqueous solution of **6′** resulted in the immediate formation of the corresponding salts **8**–**10**. Interestingly, while compounds **8**–**10** are insoluble in water, the TFA salt **6** or analogous chloride **6′** are highly soluble. These facts make our novel 1,2,4-selenodiazolium salts promising for selective anion precipitation purposes.

**Figure 4:**
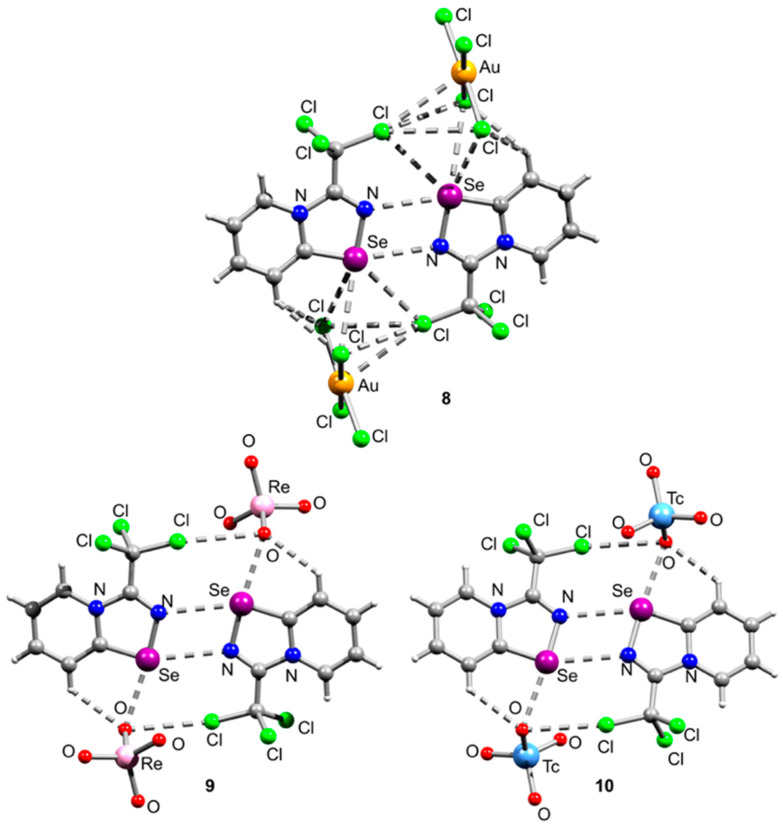
Ball-and-stick representations of the crystal structures of **8**–**10**. Grey and light-grey spheres represent carbon and hydrogen, respectively.

Compounds **8**–**10** were recrystallized from MeOH, and their structures were confirmed by the X-ray structural analysis (Figure 4).

The salts **8**–**10** formed exclusively 2Se–2N squares in the solid state (Figure 4). Switching from the chloride to AuCl_4_^−^, ReO_4_^−^, or TcO_4_^−^ did not result in the rupture of supramolecular dimers with two antiparallel Se⋯N ChB interactions. Moreover, 1,2,4-selenodiazolium cations in the dimers of **9** and **10** were interconnected by the XB between the XB donating chlorine atom of the heterocycle and AuCl_4_^−^, ReO_4_^−^, or TcO_4_^−^ anion (Figure 4).

**Figure 5:**
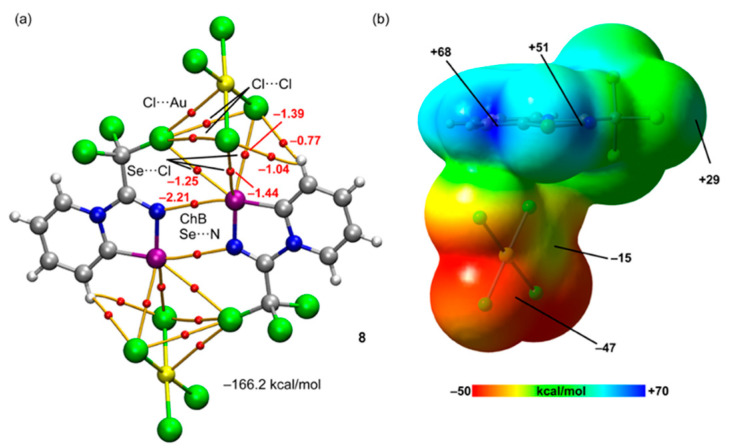
(**a**) QTAIM analysis of compound **8**. Only intermolecular bond CPs and bond paths are represented. The interaction energies of the ChB and HB contacts are indicated next to the bond CPs (small red spheres) in kcal/mol. (**b**) MEP surface of **8** using the 0.001 a.u. isodensity value. The MEP at selected points of the surface are indicated in kcal/mol.

For compound **8**, we have analyzed the possible co-existence of Cl···Cl and Cl···Au contacts in addition to the ChB and HBs. The QTAIM analysis of the tetrameric assembly is represented in Figure 5. It demonstrates the presence of an intricate combination of interactions, including two symmetrically equivalent Se···N ChBs that are the strongest ones, connecting the five membered rings. The analysis also discloses three Se···Cl contacts, two involving the tetrachloroaurate anion and one the trichloromethyl group. The energies of these ChBs are similar, ranging from −1.25 to −1.44 kcal/mol. Two additional C–H···Cl contacts connect the AuCl_4_^−^ anion to the cation. It is interesting to highlight the presence of three bond CPs and bond paths connecting one Cl atom of the trichloromethyl group to the AuCl_4_^−^, confirming the existence of Cl···Cl and Cl···Au contacts. To further analyze these contacts, we have computed the molecular electrostatic potential (MEP) surface of compound **8**, which is represented in Figure 5b. It reveals the typical σ-holes at the Se-atoms with MEP values of +68 and +51 kcal/mol. Moreover, a σ-hole is also present at the extension of the C–Cl bonds of the trichloromethyl group (+29 kcal/mol). The MEP minimum value is located at the AuCl_4_^–^ (chlorine belt), thus explaining the formation of the Cl···Cl contacts between the counterions. Moreover, the MEP value is also negative at the Au-atoms, thus revealing that the Cl···Au contact observed in **8** is also electrostatically favored.

**Figure 6:**
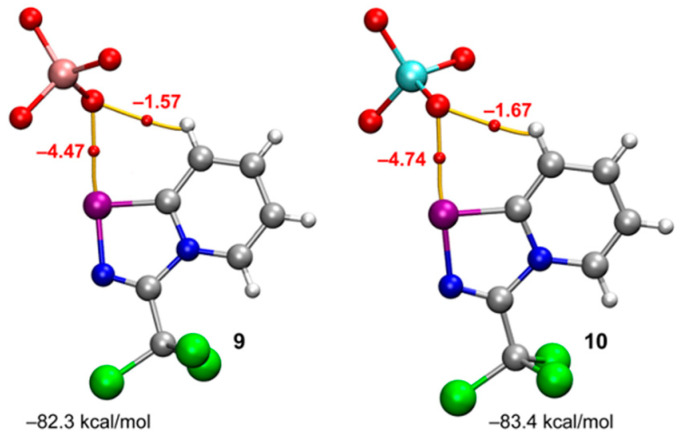
QTAIM analyses of compounds **9** and **10**. Only intermolecular bond CPs and bond paths are represented. The interaction energies of the ChB and HB contacts are indicated next to the bond CPs (small red spheres) in kcal/mol.

Moreover, for compounds **9** and **10**, we have compared the strength of the Se···O contacts. It can be observed (Figure 6) that they are weaker (−4.47 and −4.74 kcal/mol for **9** and **10**, respectively) than those of compounds **5** and **6**, due to the lower nucleophilicity of ReO_4_^−^ or TcO_4_^−^ anions compared to TFA. The energetic results gathered in Figure 6 show that the metal (Re or Tc) has little influence on the ChB and HB energies. The ion-pair interactions are large and negative (−82.3 and −83.4 kcal/mol for **9** and **10**, respectively) and smaller than those observed for **5** and **6**, in line with the ChB and HB energies.

**Figure 7:**
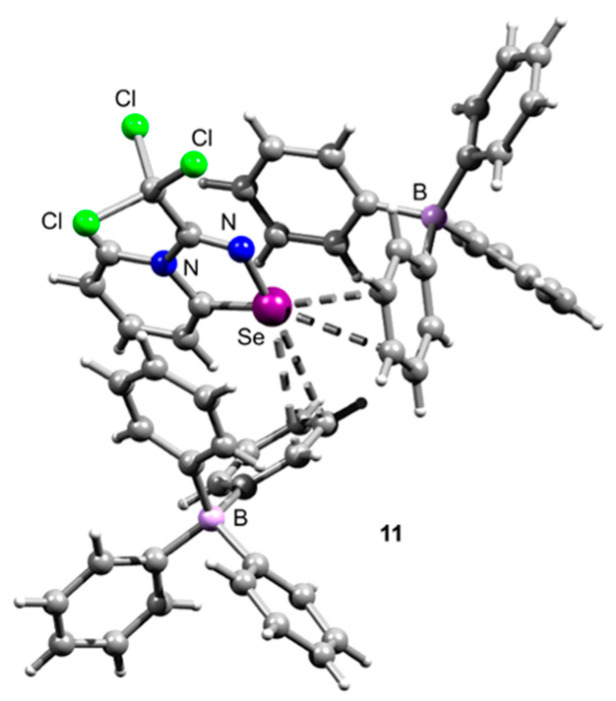
Ball-and-stick representation of the crystal structure of **11** demonstrating ChB interactions between the selenodiazolium and two neighboring tetraphenylborates. Grey and light-grey spheres represent carbon and hydrogen, respectively.

Further, we were interested in how the cation of **6′** would bind tetraphenylborate. The addition of the saturated MeOH solution of NaBPh_4_ to **6′** in MeOH resulted in the precipitation of the yellow crystals of **11** (Figure 7).

**Figure 8:**
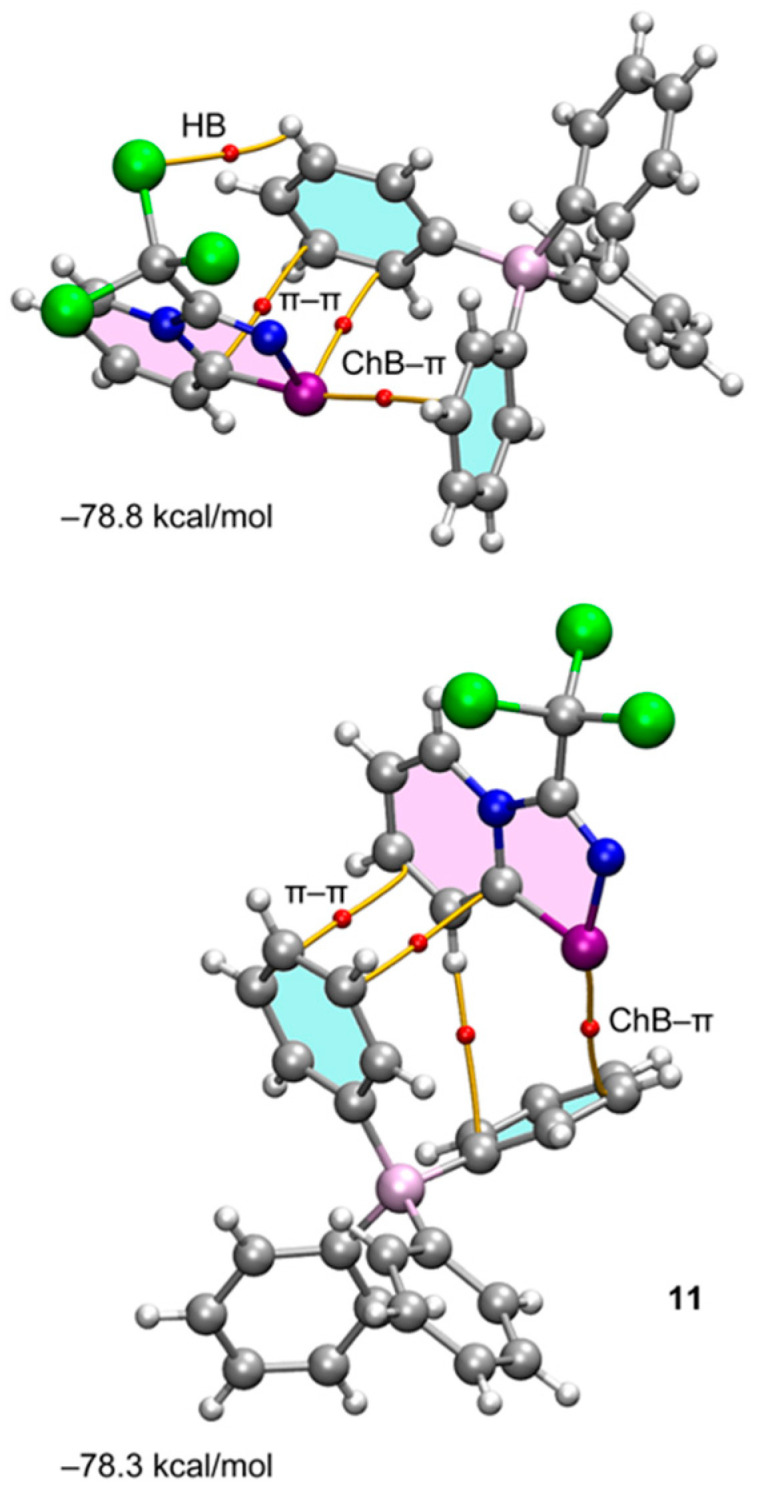
QTAIM analyses of two dimers of compound **11**. Only intermolecular bond CPs and bond paths are represented.

Interestingly, introduction of BPh_4_^–^ anion resulted in the rupture of 2Se–2N squares. The two σ-holes of selenodiazolium cation were involved in ChB–π interactions with the phenyls of BPh_4_^−^ anion. It should be noted that chalcogen–π interactions are a bonding motif found in biological systems, such as proteins [36]. Both ChB–π interactions highlighted in Figure 7 were analyzed theoretically. The QTAIM results (see Figure 8) corroborate the presence of the ChB–π interactions that are characterized by bond CPs and bond paths connecting the Se-atoms to C-atoms of the six membered rings. The formation of such assemblies was further assisted by π–π and C–H···Cl interactions, as revealed by the QTAIM analysis. The dimerization energies are −78.8 and −78.3 kcal/mol for both binding modes, which are similar to the ion-pair energies obtained for compounds **9** and **10**, thus suggesting that the ChB–π and π–π combined are almost equivalent to the ChB and HBs formed by the tetrahedral ReO_4_^−^ and TcO_4_^−^ anions.

**Figure 9:**
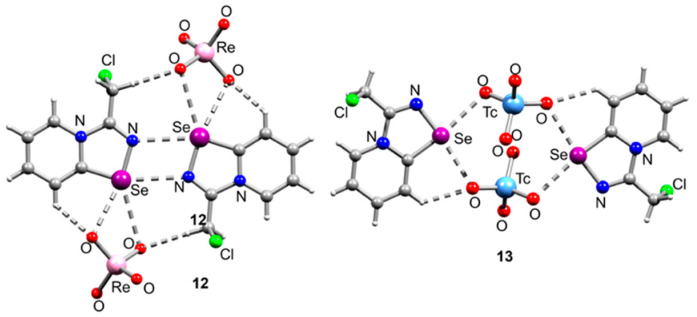
Ball-and-stick representation of the crystal structure of **12** and **13**, demonstrating supramolecular dimerization via ChB interactions.

Further, we were interested in how the substituent by the selenodiazolium core (which derives from a nitrile) would affect the self-assembly of salts, which contain ReO_4_^−^ or TcO_4_^−^ anions. The compounds **12** and **13,** which were derived from chloroacetonitrile, were simply prepared in the same way as **9** and **10**. Surprisingly, switching from the CCl_3_ to CH_2_Cl group had a noticeable impact on the self-assembly of the compound in the solid state (Figure 9).

Compound **12** formed Se_2_N_2_ squares in the solid state in the same fashion as **9** and **10**. However, it had several distinctive features. The ReO_4_^−^ anion was involved in a bifurcated ChB interaction with the Se center via two O atoms (Figure 9). In contrast, structurally similar compounds **9** and **10** featured a terminal coordination of the ReO_4_^−^ and TcO_4_^−^ anions (Figure 4). Another interesting structural peculiarity of **12** was the presence of H···O HB interactions between the ReO_4_^−^ anion and α-H atom of the substituent by the selenodiazolium core (Figure 9) in the solid state. H···O HB was preferential here over a potential Cl···O XB interaction. It should be noted that selenodiazolium salts, which contained the same cation but the Cl or Br anions, formed similar dimers, which exhibited (*N*.*B*.) Cl···Cl or Cl···Br XB interactions [16], but not H···Cl or H···Br HB.

Compound **13** exhibited even a more distinctive pattern in the crystal (Figure 9). In contrast to **9**, **10**, or **12**, it did not exhibit Se_2_N_2_ squares but formed dimers via four Se···O ChB interactions with two bridging TcO_4_^−^ anions (Figure 9).

**Figure 10:**
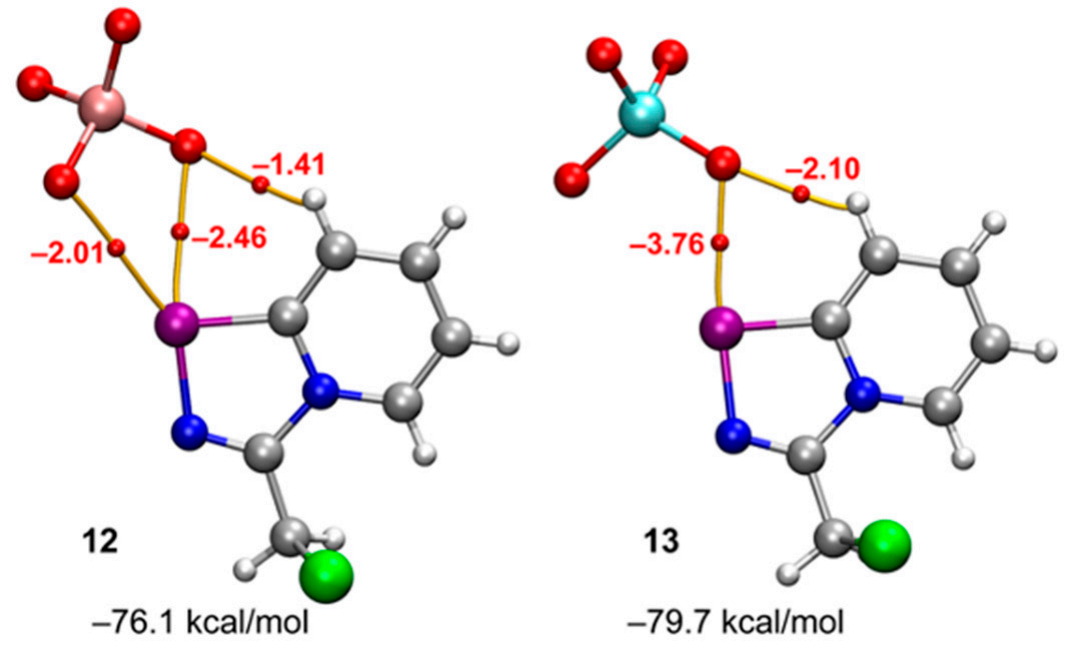
QTAIM analyses of compounds **12** and **13**. Only intermolecular bond CPs and bond paths are represented. The energies of the ChB and HB contacts are indicated next to the bond CPs (small red spheres) in kcal/mol.

Cation-anion interactions in **12** and **13** were further studied theoretically. Figure 10 shows the QTAIM analyses of the ion-pair interactions of compounds **12** and **13**, evidencing the bifurcated nature of the ChB in **12**, which is stronger (−4.47 kcal/mol, sum of both CPs) than the Se···O ChB in **13**. This is mostly compensated by the HB that is stronger in compound **12**. The ChBs in **12** and **13** are weaker than those of compounds **9** and **10**, due to the stronger electron withdrawing effect of the CCl_3_ group. Regarding the ion-pair dimerization energies, it is larger in compound **13** (−79.7 kcal/mol) than **12** (−76.1 kcal/mol), and both are similar to the ChB–π dimers represented in Figure 8.

**Figure 11:**
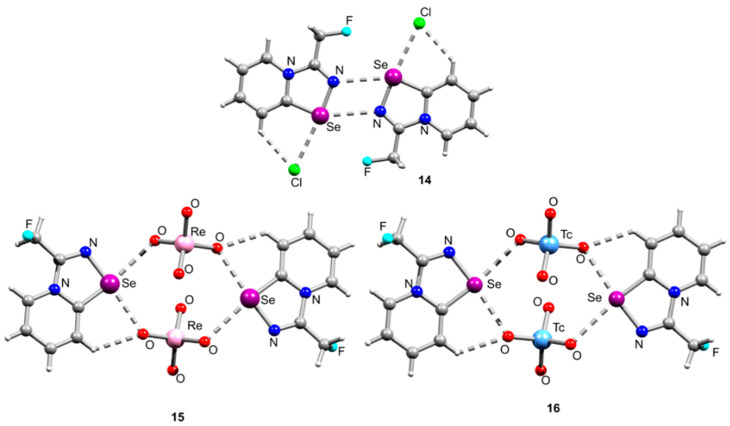
Ball-and-stick representation of crystal structures **14**–**16** demonstrating supramolecular dimerization via various ChB interactions.

Finally, we compared the impact of switching from the Cl to the F in haloacetonitrile on the self-assembly of corresponding selenodiazolium salts. For this reason, 2-pyridylselenylchloride was coupled with fluoroacetonitrile to give novel adduct **14** (Figure 11). X-ray analysis showed that **14** also formed Se_2_N_2_ dimers in the solid state in a similar fashion to what we observed for several other selenodiazolium salts [15,16].

The F-decorated selenodiazolium cation also formed water-insoluble salts **15** and **16** with perrhenate or pertechnetate, correspondingly. Both **15** and **16** formed dimers via four Se···O ChB interactions with two bridging ReO_4_^−^ or TcO_4_^−^ anions (Figure 11).

**Figure 12:**
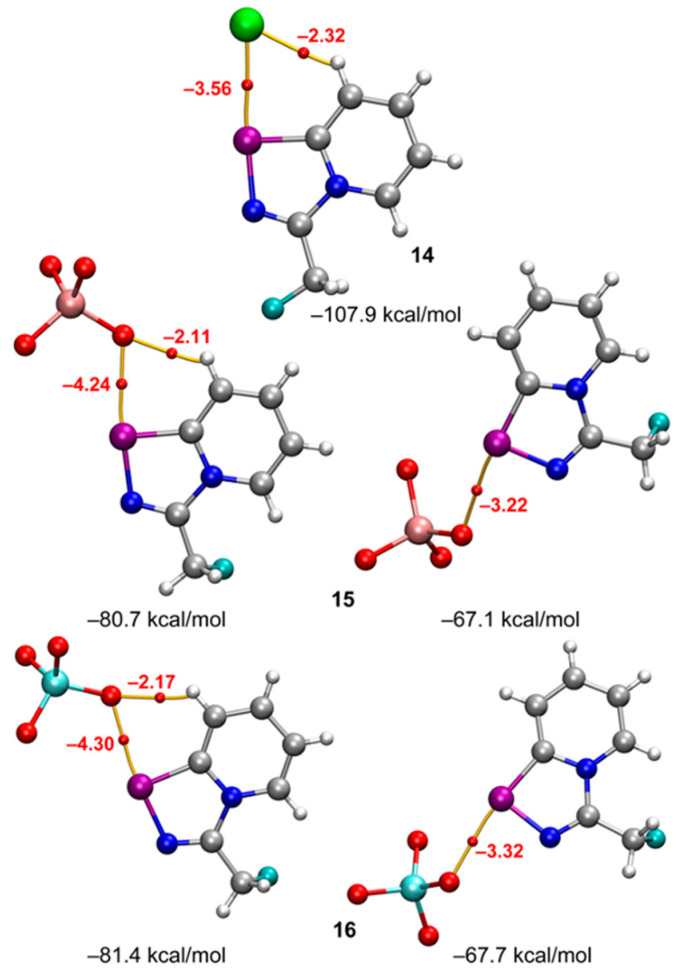
QTAIM analyses of compounds **14**–**17**. Only intermolecular bond CPs and bond paths are represented. The interaction energies of the ChB and HB contacts are indicated next to the bond CPs (small red spheres) in kcal/mol.

The QTAIM analyses of the ion-pair dimers of compounds **14**–**16** are represented in Figure 12. For compounds **15** and **16**, where the anion is bridging the five membered selenodiazolium rings (Figure 11), we have analyzed both types of Se···O ChBs, where the O-atom is located opposite the Se–N or Se–C bond.

The energies of the ChBs opposite the Se–N bond are stronger than those opposite the C, in line with the MEP analysis of Figure 5b, which evidences a more intense σ-hole opposite the Se–N bond. The HBs energies are quite similar in the three complexes. The ion-pair energies of complexes **15** and **16** show that the dimers, where the anion is opposite the C–N bond, are significantly more favored than those where it is opposite the Se–C bond. Finally, in compound **14**, the ion-pair energy is significantly larger (−107.9 kcal/mol) due to the higher nucleophilicity of the Cl-atom (in the other anions, the negative charge is shared by all the O-atoms).

## 3. Materials and Methods

**General remarks.** All manipulations were carried out in air. All the reagents used in this study were obtained from the commercial sources (Aldrich, TCI-Europe, Strem, ABCR). Commercially available solvents were purified by conventional methods and distilled immediately prior to use. NMR spectra were recorded on a Bruker Avance Neo (^1^H: 700 MHz); chemical shifts (*δ*) were given in ppm, coupling constants (*J*) in Hz. C, H, and N elemental analyses were carried out on a Euro EA 3028HT CHNS/O analyzer. Mass-spectra were obtained on a Bruker micrOTOF spectrometer equipped with an electrospray ionization (ESI) source; a MeOH, CH_2_Cl_2_, or MeOH/CH_2_Cl_2_ mixture was used as a solvent. 2-Pyridylselenylbromide and di(2-pyridyl)diselenide were prepared as reported earlier [37]. 2-Pyridylselenylchloride was obtained by the method reported earlier [38].

**X-ray crystal structure determination.** The single-crystal X-ray diffraction data for 6, 8, 11, and 14 were collected on the ‘RSA’ beamline of the National Research Center ‘Kurchatov Institute’ (Moscow, Russian Federation) using a Rayonix SX165 CCD detector. A total of 720 images for two different orientations in the case of each crystal were collected using an oscillation range of 1.0° and *φ* scan mode. The data were indexed and integrated using the utility *iMOSFLM* in the CCP4 program [39] and then scaled and corrected for absorption using the *Scala* program [40]. The single-crystal X-ray diffraction data for 7, 8, 9, 10, 12, and 14 were collected on a three-circle Bruker D8 Venture (*Kurnakov Institute of General and Inorganic Chemistry*, *Russian Academy of Sciences*) or Bruker D8 QUEST PHOTON-III CCD (*Zelinsky Institute of Organic Chemistry*, *Russian Academy of Sciences*) diffractometers using *φ* and *ω* scan mode. The crystal structures of 10, 13, and 16 were determined by X-ray structural analysis using an automatic four-circle area-detector diffractometer Bruker KAPPA APEX II with MoKα radiation (*Frumkin Institute of Physical Chemistry and Electrochemistry*, *Russian Academy of Sciences*). The data were indexed and integrated using the SAINT program [41] and then scaled and corrected for absorption using the SADABS program [42]**.** Structures 10, 13, and 16 were solved by using the SHELXT-2018/2 program [43]. All other structures were determined by direct methods and refined by the full-matrix least squares technique on *F*^2^ with anisotropic displacement parameters for non-hydrogen atoms. The hydrogen atom of the OH-group in 11 was localized in the difference-Fourier map and refined isotropically with fixed displacement parameters (*U*_iso_(H) = 1.5*U*_eq_(O)). The other hydrogen atoms in all compounds were placed in calculated positions and refined within a riding model with fixed isotropic displacement parameters (*U*_iso_(H) = 1.5*U*_eq_(C) for the CH_3_-groups and 1.2*U*_eq_(C) for the other groups). All calculations were carried out using the SHELXTL program [44]. Crystallographic data for all investigated compounds were deposited with the Cambridge Crystallographic Data Center, CCDC 2174270–2174282. Copies of this information may be obtained free of charge from the Director, CCDC, 12 Union Road, Cambridge CHB2 1EZ, UK (Fax: +44-1223-336033; e-mail: deposit@ccdc.cam.ac.uk or www.ccdc.cam.ac.uk).

**Computational details.** DFT calculations were carried out on the X-ray coordinates (SI) at the ωB97X-D3/def2-TZVPP level of theory using ORCA 4.2.1 [45,46]. MultiWFN 3.8 [47] was used to perform the QTAIM analyses to obtain critical points of electron density, corresponding bond paths, and characteristics and to plot reduced density gradient plots. Natural bond orbital theory (NBO) calculations were made by means of NBO 5.9 suite [48]. Molecular electrostatic potential maps were visualized using VMD 1.9.3 [49].

**Synthesis of compounds 4−16.**

**Synthesis of 4.** Bis(trifluoroacetoxy)iodo)benzene (PIFA) (30.0 mg, 0.07 mmol) in Et_2_O (1 mL) was added to 2,2′-dipyridyldiselenide (21.9 mg, 0.07 mmol) in Et_2_O (1 mL), and the reaction mixture was left without stirring for 3 h. After that, a solution was decanted from colorless crystalline precipitate, which was washed with Et_2_O (3 × 1 mL) and dried under vacuum. Yield: 16.2 mg (42%). Elem. anal. calcd for C_7_H_4_F_3_NO_2_Se: C, 31.13; H, 1.49; N, 5.19. Found: C 31.28; H 1.53; N 4.95. ^1^H NMR (700 MHz, CD_3_OD) *δ* 8.71 (1H, d, *J* = 4.7 Hz, H6), 8.13 (1H, td, *J* = 7.7, 1.7 Hz, H4), 8.08 (1H, d, *J* = 7.8 Hz, H5), 7.63 (1H, ddd, *J* = 7.4, 4.7, 1.1 Hz, H3), ^13^C{^1^H} NMR (176 MHz, CD_3_OD) *δ* 166.9 (Py-C2), 151.6 (Py-C6), 139.7 (Py-C4), 128.4 (Py-C5), 122.2 (Py-C3), *C*F_3_*C*(O)O signals were not observed. ^19^F NMR (659 MHz, CD_3_OD) *δ* –76.94.

**Synthesis of 5.** The solution of PIFA (29.3 mg, 0.07 mmol) and acetonitrile (100 µL) in Et_2_O (0.5 mL) was added to 2,2′-dipyridyldiselenide (20.8 mg, 0.07 mmol) in Et_2_O (0.5 mL), and the reaction mixture was left without stirring for 3 h. After that, a solution was decanted from colorless crystalline precipitate, which was washed with Et_2_O (3 × 1 mL) and dried under vacuum. Yield: 24 mg (56%). ^1^H NMR (700 MHz, D_2_O) *δ* 9.34 (d, *J* = 6.8 Hz, 1H), 8.80 (d, *J* = 8.7 Hz, 1H), 8.41 (t, *J* = 8.4 Hz, 1H), 8.04 (t, *J* = 7.0 Hz, 1H), 3.01 (s, 3H). ^13^C{^1^H} NMR (176 MHz, D_2_O) *δ* 167.5, 162.9 (q, *J* = 35.4 Hz), 156.2, 139.3, 136.2, 125.6, 122.9, 116.3 (q, *J* = 291.6 Hz), 17.2 (CH_3_). ^19^F NMR (659 MHz, D_2_O) *δ* −75.60.

**Synthesis of 6.** The solution of PIFA (30.0 mg, 0.07 mmol) and trichloroacetonitrile (100 µL) in Et_2_O (0.5 mL) was added to 2,2′-dipyridyldiselenide (21.8 mg, 0.07 mmol) in Et_2_O (0.5 mL), and the reaction mixture was left without stirring for 12 h. After that, a solution was decanted from colorless crystalline precipitate, which was washed with Et_2_O (3 × 1 mL) and dried under vacuum. Yield: 37 mg (64%). ^1^H NMR (700 MHz, D_2_O) *δ* 9.95 (d, *J* = 7.0 Hz, 1H), 8.95 (d, *J* = 8.7 Hz, 1H), 8.51 (t, *J* = 8.0 Hz, 1H), 8.13 (t, *J* = 7.1 Hz, 1H). ^13^C{^1^H} NMR (176 MHz, D_2_O) *δ* 171.6, 162.3 (q, *J* = 35.4 Hz), 148.1, 140.1, 138.3, 126.4, 123.2, 116.3 (q, *J* = 291.8 Hz), 87.5. ^19^F NMR (659 MHz, D_2_O) *δ* −75.58.

**Synthesis of 7.** The solution of PIFA (45.0 mg, 0.1 mmol) and hexanenitrile (29.2 mg, 0.34 mmol) in Et_2_O (1.0 mL) was added to 2,2′-dipyridyldiselenide (32.9 mg, 0.1 mmol) in Et_2_O (1.0 mL), and the reaction mixture was left without stirring for 3 h. After that, a solution was decanted from colorless crystalline precipitate, which was washed with Et_2_O (3 × 1 mL) and dried under vacuum. Yield: 28 mg (74%). ^1^H NMR (700 MHz, D_2_O) *δ* 9.37 (d, *J* = 6.8 Hz, 1H), 8.80 (d, *J* = 8.6 Hz, 1H), 8.40 (t, *J* = 7.9 Hz, 1H), 8.02 (t, *J* = 7.0 Hz, 1H), 3.34 (t, *J* = 7.5 Hz, 2H), 2.00 (p, *J* = 7.5 Hz, 2H), 1.49 (p, *J* = 7.8, 7.4 Hz, 2H), 1.40 (h, *J* = 7.3 Hz, 2H), 0.91 (t, *J* = 7.3 Hz, 3H). ^13^C NMR (176 MHz, D_2_O) *δ* 167.7, 159.2, 139.3, 135.9, 125.8, 122.8, 116.3 (q, *J* = 291.9 Hz), 30.7, 30.3, 24.2, 21.6, 13.1. CF_3_*C*(O)O signal was not observed. ^19^F NMR (659 MHz, D_2_O) *δ* −75.61.

**Synthesis of 8.** 2-Pyridylselenyl chloride (89 µmol, 15.1 mg) was suspended in Et_2_O (4 mL), then trichloroacetonitrile (1 mL) was added, and the mixture was stirred at room temperature for 12 h. Colorless precipitate formed was filtered, dried under vacuum, and redissolved in MeOH (3 mL). Addition of the MeOH solution (100 µL) of NaAuCl_4_ (50 mg) resulted in the formation of yellow microcrystalline precipitate. Yield: 27 mg (54%). Elem. anal. calcd for C_7_H_4_AuCl_7_N_2_Se: C, 13.13; H, 0.63; N, 4.38. Found: C 13.36; H 1.11; N 4.32. ^1^H NMR (700 MHz, CD_3_OD) *δ* 9.98 (dt, *J* = 7.0 Hz, 1H, H5), 9.06 (dt, *J* = 8.7 Hz, 1H, H8), 8.55 (ddd, *J* = 8.6 Hz, 1H, H7), 8.17 (td, *J* = 7.0 Hz, 1H, H6). ^13^C{^1^H} NMR *δ* 174.3 (C3), 149.3 (C9), 141.0 (C5), 139.7 (C8), 128.3 (C7), 124.4 (C6), 89.6 (CCl_3_). MS (ESI^+^), found: 300.8592 [M–AuCl_4_]^+^; calcd for C_7_H_4_Cl_3_N_2_Se: 300.8600.

**Synthesis of 9.** 2-Pyridylselenyl chloride (260 µmol, 20 mg) and trichloroacetonitrile (1.30 mmol, 130 µL) were stirred in Et_2_O (4 mL) at room temperature for 12 h. After that, solvent was evaporated, the residue was redissolved in MeOH (3 mL), and 40 µL of perrhenic acid (70 wt%) was added. Colorless precipitate, which gradually formed, was filtered, washed with Et_2_O (3 × 3 mL), and dried under vacuum. Yield: 44 mg (76%). Elem. anal. calcd for C_7_H_4_Cl_3_N_2_O_4_ReSe: C, 15.24; H, 0.73; N, 5.08. Found: C 15.52; H 0.95; N 5.06. ^1^H NMR (700 MHz, D_2_O) *δ* 9.95 (1H, dd, *J* = 6.8, 1.2 Hz, H5), 8.95 (1H, dd, *J* = 8.6, 1.4 Hz, H8), 8.50 (1H, ddd, *J* = 8.5, 7.2, 1.1 Hz, H7), 8.13 (1H, td, *J* = 7.1, 1.4 Hz, H6). ^13^C{^1^H} NMR *δ* 171.7 (C3), 148.1 (C9), 140.1 (C5), 138.3 (C8), 129.1 (CCl_3_), 126.4 (C7), 123.1 (C6).

**Synthesis of 10.** 2-Pyridylselenyl chloride (93 µmol, 25 mg) and trichloroacetonitrile (50 µL) were stirred in Et2O (4 mL) at room temperature for 12 h. After that, solvent was evaporated, the residue was redissolved in MeOH (3 mL), 10 µL of pertechnetic acid (5 M) was added, and the mixture was left for 3 h until colorless crystals formed. Yield: 44 mg (73%).

**Synthesis of 11.** 2-Pyridylselenyl chloride (89 µmol, 14.5 mg) was suspended in Et_2_O (5 mL), then trichloroacetonitrile (1 mL) was added, and the mixture was stirred at room temperature for 12 h. Colorless precipitate formed was filtered, dried under vacuum, and redissolved in MeOH (3 mL). Addition of the saturated MeOH solution of NaBPh_4_ (100 µL) resulted in the formation of microcrystalline precipitate. Yield: 19.2 mg (72%). Elem. anal. calcd for C_31_H_24_BCl_3_N_2_Se: C, 59.99; H, 3.90; N, 4.51. Found: C 60.31; H 3.79; N 4.48. ^1^H NMR (700 MHz, D_2_O) *δ* 9.76 (dd, *J* = 6.9 Hz, 1H, H5), 9.13 (dt, *J* = 8.7 Hz, 1H, H8), 8.46 (ddd, *J* = 8.5 Hz, 1H, H7), 8.10 (td, *J* = 7.0 Hz, 1H, H6). ^13^C{^1^H} NMR *δ* 173.1 (C3), 163.4 (dd, J = 98.6, 49.3 Hz, ipso-C from Ph’s), 146.7 (C9), 139.5 (C5), 137.9 (C8), 135.6 (o-C from Ph’s), 127.5 (C7), 125.3 (m-C from Ph’s), 123.2 (C6), 121.5 (p-C from Ph’s), 88.3 (CCl_3_).

**Synthesis of 12.** 2-Pyridylselenyl chloride (104 µmol, 20 mg) and chloroacetonitrile (312 µmol, 31 µL) were stirred in Et_2_O (4 mL) at room temperature for 12 h. After that, solvent was evaporated, the residue was redissolved in MeOH (3 mL), and 40 µL of perrhenic acid (70 wt%) was added. Colorless precipitate, which gradually formed, was filtered, washed with Et_2_O (3 × 3 mL), and dried under vacuum. Yield: 37 mg (74%). Elem. anal. calcd for C_7_H_6_ClN_2_O_4_ReSe: C, 17.42; H, 1.25; N, 5.80. Found: C 17.45; H 1.21; N 5.83. ^1^H NMR (600 MHz, D_2_O) *δ* 9.51 (1H, d, J = 6.8 Hz, H5), 8.86 (1H, d, *J* = 8.7 Hz, H8), 8.46 (1H, t, J = 8.0 Hz, H7), 8.09 (1H, t, J = 7.0 Hz, H6), 5.35 (2H, s, CH_2_). ^13^C{^1^H} NMR *δ* 168.7 (C3), 152.9 (C9), 139.8 (C5), 136.4 (C8), 126.1 (C7), 123.3 (C6), 37.7 (CH_2_).

**Synthesis of 13.** 2-Pyridylselenyl chloride (93 µmol, 25 mg) and chloroacetonitrile (312 µmol, 31 µL) were stirred in Et_2_O (4 mL) at room temperature for 12 h. After that, solvent was evaporated, the residue was redissolved in MeOH (3 mL), 10 µL of pertechnetic acid (5 M) was added, and the mixture was left for 3 h until colorless crystals formed. Yield: 36 mg (70%).

**Synthesis of 14.** 2-Pyridylselenyl chloride (500 µmol, 100 mg) and fluoroacetonitrile (10 µmol, 60 mg) were stirred in Et_2_O (4 mL) at room temperature for 12 h. Colorless precipitate, which gradually formed, was filtered, washed with Et_2_O (3 × 3 mL), and dried under vacuum. Yield: 110 mg (78%). Elem. anal. calcd for C_7_H_6_ClFN_2_Se: C, 33.42; H, 2.40; N, 11.14. Found: C 33.48; H 2.41; N 11.18. ^1^H NMR (700 MHz, D_2_O) *δ* 9.38 (d, *J* = 6.8 Hz, 1H), 8.79 (d, *J* = 8.7 Hz, 1H), 8.37 (t, *J* = 8.0 Hz, 1H), 7.99 (t, *J* = 7.0 Hz, 1H), 5.99 (d, *J* = 45.9 Hz, 2H). ^13^C{^1^H} NMR (176 MHz, D_2_O) *δ* 168.6, 152.3 (d, *J* = 19.8 Hz), 140.0, 136.3, 126.1, 123.4, 78.0 (d, *J* = 172.4 Hz). ^19^F NMR (659 MHz, D_2_O) *δ* 212.70 (t, *J* = 45.9 Hz).

**Synthesis of 15.** 2-Pyridylselenyl chloride (520 µmol, 100 mg) and fluoroacetonitrile (10 µmol, 60 mg) were stirred in Et_2_O (4 mL) at room temperature for 12 h at room temperature. After that, solvent was evaporated, the residue was redissolved in MeOH (3 mL), and 100 µL of perrhenic acid (70 wt %) was added. Colorless precipitate, which gradually formed, was filtered, washed with Et_2_O (3 × 3 mL), and dried under vacuum. Yield: 213 mg (81%). ^1^H NMR (700 MHz, D_2_O) *δ* 9.38 (d, *J* = 6.8 Hz, 1H), 8.77 (d, *J* = 8.7 Hz, 1H), 8.37 (t, *J* = 8.0 Hz, 1H), 7.99 (t, *J* = 7.0 Hz, 1H), 5.99 (d, *J* = 45.9 Hz, 2H). ^13^C NMR (176 MHz, D_2_O) *δ* 168.5, 152.4, 140.0, 136.2, 126.0, 123.4, 78.0 (d, *J* = 172.1 Hz).

**Synthesis of 16.** 2-Pyridylselenyl chloride (93 µmol, 25 mg) and fluoroacetonitrile (50 µL) were stirred in Et_2_O (4 mL) at room temperature for 12 h. After that, solvent was evaporated, the residue was redissolved in MeOH (3 mL), 10 µL of pertechnetic acid (5 M) was added, and the mixture was left for 3 h until colorless crystals formed. Yield: 37 mg (73%).

## 4. Conclusions

In conclusion, we prepared and structurally characterized a series of 1,2,4-selenodiazolium salts with various anions. Trifluoroacetate derivatives were obtained via novel Se–Se bond scission reaction of 2,2′-dipyridyl diselenide with bis(trifluoroacetoxy)iodo)benzene in the presence of corresponding nitriles. The reactive 2-pyridylselenyl trifluoroacetate was also isolated and structurally characterized with the help of the X-ray single crystal analysis, which revealed that **4** forms supramolecular dimers in the solid state via a pair of equivalent Se⋯N ChB interactions. The dimers of **4** featured short and strong ChB contacts Se⋯O (21.5 kcal/mol) and Se⋯N (24.9 kcal/mol) and terminal binding with trifluoroacetate anion. In contrast, selenodiazolium cations bind trifluoroacetate via a pair of “chelating” Se⋯O and H⋯O non-covalent interactions, which is geometrically allowed.

1,2,4-Selenodiazolium trifluoroacetates or halides show good solubility in water. In contrast, (AuCl_4_)^−^, (ReO_4_)^−^, or (TcO_4_)^−^ derivatives immediately precipitate from aqueous solutions. This fact makes selenodiazolium cations attractive for anion precipitation purposes.

The binding of AuCl_4_^−^, ReO_4_^−^, and TcO_4_^−^ with model pyridine-fused 1,2,4-selenodiazolium cations, carrying halogen substituents in the core, was further regarded. To the best of our knowledge, this is the first study to regard perrhenate and pertechnetate as acceptors in ChB interactions. In these supramolecular complexes, selenodiazolium cations act as polyfunctional ChB, HB, and XB donors, as well as ChB acceptors. Regardless of the nature of the anion, the combination of directional Se⋯O ChB and H⋯O HB dictates the position of the anion in the supramolecular complex. In **8**, featuring the AuCl_4_^−^ anion, the presence of intricate combination of Cl···Cl, H···Cl, Se···Cl, and Cl···Au interactions was observed, involving the tetrachloroaurate anion and the trichloromethyl group. A more detailed and systematic study of selenodiazolium complexes with AuCl_4_^−^ is required and will be published soon by our group.

In **8**, selenodiazolium cations form supramolecular dimers via two antiparallel Se···N interactions. Switching from AuCl_4_^−^ to ReO_4_^−^ and TcO_4_^−^ did not result in dimers rupture. The ReO_4_^−^ or TcO_4_^−^ anion again formed a bifurcated Se⋯O and H⋯O position. However, switching to BPh_4_^−^ did result in the dimer rupture due to the formation of stronger Se⋯π interactions. Small structural variations (switching from CCl_3_ to CH_2_Cl or CH_2_F) in the aliphatic substituent of the cation did not have any dramatic influence on the overall situation. However, for **13**, **15**, and **16**, we observed 2Se–2N rupture and supramolecular dimerization via two bridging ReO_4_^−^ or TcO_4_^−^ anions.

The structural and computational results presented here will be useful for further developments in anion recognition, and precipitation involving more elaborated water-soluble cationic 1,2,4-selenodiazoles, designed for specific anions, is currently underway in our laboratory and will be reported in a due course.

## References

[1] Langton M.J., Robinson S.W., Marques I., Félix V., Beer P.D. (2014). Halogen bonding in water results in enhanced anion recognition in acyclic and rotaxane hosts. Nat. Chem..

[2] Taylor M.S. (2020). Anion recognition based on halogen, chalcogen, pnictogen and tetrel bonding. Coord. Chem. Rev..

[3] Luecke H., Quiocho F.A. (1990). High specificity of a phosphate transport protein determined by hydrogen bonds. Nature.

[4] Beer P.D., Gale P.A. (2001). Anion Recognition and Sensing: The State of the Art and Future Perspectives. Angew. Chem. Int. Ed..

[5] Evans N.H., Beer P.D. (2014). Advances in Anion Supramolecular Chemistry: From Recognition to Chemical Applications. Angew. Chem..

[6] Langton M.J., Serpell C.J., Beer P.D. (2016). Anion Recognition in Water: Recent Advances from a Supramolecular and Macromolecular Perspective. Angew. Chem..

[7] Mahmudov K.T., Kopylovich M.N., Guedes da Silva M.F.C., Pombeiro A.J.L. (2017). Non-covalent interactions in the synthesis of coordination compounds: Recent advances. Coord. Chem. Rev..

[8] Ho P.C., Wang J.Z., Meloni F., Vargas-Baca I. (2020). Chalcogen bonding in materials chemistry. Coord. Chem. Rev..

[9] Tskhovrebov A.G., Novikov A.S., Kritchenkov A.S., Khrustalev V.N., Haukka M. (2020). Attractive halogen···halogen interactions in crystal structure of trans-dibromogold(III) complex. Z. Krist. Cryst. Mater..

[10] Shikhaliyev N.G., Maharramov A.M., Bagirova K.N., Suleymanova G.T., Tsyrenova B.D., Nenajdenko V.G., Novikov A.S., Khrustalev V.N., Tskhovrebov A.G. (2021). Supramolecular organic frameworks derived from bromoaryl-substituted dichlorodiazabutadienes via Cl···Br halogen bonding. Mendeleev Commun..

[11] Nenajdenko V.G., Shikhaliyev N.G., Maharramov A.M., Bagirova K.N., Suleymanova G.T., Novikov A.S., Khrustalev V.N., Tskhovrebov A.G. (2020). Halogenated Diazabutadiene Dyes: Synthesis, Structures, Supramolecular Features, and Theoretical Studies. Molecules.

[12] Khrustalev V.N., Savchenko A.O., Zhukova A.I., Chernikova N.Y., Kurykin M.A., Novikov A.S., Tskhovrebov A.G. (2021). Attractive fluorine···fluorine interactions between perfluorinated alkyl chains: A case of perfluorinated Cu(II) diiminate Cu[C2F5-C(NH)-CF=C(NH)-CF3]2. Z. Krist. Cryst. Mater..

[13] Tskhovrebov A.G., Novikov A.S., Tupertsev B.S., Nazarov A.A., Antonets A.A., Astafiev A.A., Kritchenkov A.S., Kubasov A.S., Nenajdenko V.G., Khrustalev V.N. (2021). Azoimidazole Gold(III) Complexes: Synthesis, Structural Characterization and Self-Assembly in the Solid State. Inorg. Chim. Acta.

[14] Tskhovrebov A.G., Vasileva A.A., Goddard R., Riedel T., Dyson P.J., Mikhaylov V.N., Serebryanskaya T.V., Sorokoumov V.N., Haukka M. (2018). Palladium(II)-Stabilized Pyridine-2-Diazotates: Synthesis, Structural Characterization, and Cytotoxicity Studies. Inorg. Chem..

[15] Khrustalev V.N., Grishina M.M., Matsulevich Z.V., Lukiyanova J.M., Borisova G.N., Osmanov V.K., Novikov A.S., Kirichuk A.A., Borisov A.V., Solari E. (2021). Novel cationic 1,2,4-selenadiazoles: Synthesis via addition of 2-pyridylselenyl halides to unactivated nitriles, structures and four-center Se⋯N contacts. Dalt. Trans..

[16] Grudova M.V., Khrustalev V.N., Kubasov A.S., Strashnov P.V., Matsulevich Z.V., Lukiyanova J.M., Borisova G.N., Kritchenkov A.S., Grishina M.M., Artemjev A.A. (2022). Adducts of 2-Pyridylselenenyl Halides and Nitriles as Novel Supramolecular Building Blocks: Four-Center Se···N Chalcogen Bonding versus Other Weak Interactions. Cryst. Growth Des..

[17] Buslov I.V., Novikov A.S., Khrustalev V.N., Grudova M.V., Kubasov A.S., Matsulevich Z.V., Borisov A.V., Lukiyanova J.M., Grishina M.M., Kirichuk A.A. (2021). 2-Pyridylselenenyl versus 2-Pyridyltellurenyl Halides: Symmetrical Chalcogen Bonding in the Solid State and Reactivity towards Nitriles. Symmetry.

[18] Grudova M.V., Kubasov A.S., Khrustalev V.N., Novikov A.S., Kritchenkov A.S., Nenajdenko V.G., Borisov A.V., Tskhovrebov A.G. (2022). Exploring Supramolecular Assembly Space of Cationic 1,2,4-Selenodiazoles: Effect of the Substituent at the Carbon Atom and Anions. Molecules.

[19] Lieser K.H. (1993). Technetium in the Nuclear Fuel Cycle, in Medicine and in the Environment. Radiochim. Acta.

[20] Kooyman T. (2021). Current state of partitioning and transmutation studies for advanced nuclear fuel cycles. Ann. Nucl. Energy.

[21] Zhang Y., Su R., Chen X., Ren C., Lv Y., Mo D., Liu M., Yan S. (2019). The development status of PUREX process for nuclear fuel reprocessing from an insight from PATENTS. J. Radioanal. Nucl. Chem..

[22] Bader R.F.W. (1990). Atoms in Molecules: A Quantum Theory.

[23] Mata I., Alkorta I., Espinosa E., Molins E. (2011). Relationships between interaction energy, intermolecular distance and electron density properties in hydrogen bonded complexes under external electric fields. Chem. Phys. Lett..

[24] Bauzá A., Frontera A. (2020). Halogen and Chalcogen Bond Energies Evaluated Using Electron Density Properties. ChemPhysChem.

[25] Kritchenkov A.S., Bokach N.A., Starova G.L., Kukushkin V.Y. (2012). A palladium(II) Center activates nitrile ligands toward 1,3-dipolar cycloaddition of nitrones substantially more than the corresponding platinum(II) center. Inorg. Chem..

[26] Allen F.H., Kennard O., Watson D.G., Brammer L., Orpen A.G., Taylor R. (1987). Tables of bond lengths determined by X-ray and neutron diffraction. Part 1. Bond lengths in organic compounds. J. Chem. Soc. Perkin Trans. 2.

[27] Tskhovrebov A.G., Solari E., Scopelliti R., Severin K. (2014). Reactions of grignard reagents with nitrous oxide. Organometallics.

[28] Tskhovrebov A.G., Bokach N.A., Haukka M., Kukushkin V.Y. (2009). Different routes for amination of platinum(II)-bound cyanoguanidine. Inorg. Chem..

[29] Liu Y., Varava P., Fabrizio A., Eymann L.Y.M., Tskhovrebov A.G., Planes O.M., Solari E., Fadaei-Tirani F., Scopelliti R., Sienkiewicz A. (2019). Synthesis of aminyl biradicals by base-induced Csp3–Csp3 coupling of cationic azo dyes. Chem. Sci..

[30] Mikhaylov V.N., Sorokoumov V.N., Liakhov D.M., Tskhovrebov A.G., Balova I.A. (2018). Polystyrene-supported acyclic diaminocarbene palladium complexes in Sonogashira cross-coupling: Stability vs. catalytic activity. Catalysts.

[31] Tskhovrebov A.G., Solari E., Scopelliti R., Severin K. (2013). Insertion of zerovalent nickel into the N-N bond of N-heterocyclic-carbene- activated N2O. Inorg. Chem..

[32] Mikhaylov V.N., Sorokoumov V.N., Novikov A.S., Melnik M.V., Tskhovrebov A.G., Balova I.A. (2020). Intramolecular hydrogen bonding stabilizes trans-configuration in a mixed carbene/isocyanide PdII complexes. J. Organomet. Chem..

[33] Astafiev A.A., Repina O.V., Tupertsev B.S., Nazarov A.A., Gonchar M.R., Vologzhanina A.V., Nenajdenko V.G., Kritchenkov A.S., Khrustalev V.N., Nadtochenko V.N. (2021). Unprecedented Coordination-Induced Bright Red Emission from Group 12 Metal-Bound Triarylazoimidazoles. Molecules.

[34] Kritchenkov A.S., Luzyanin K.V., Bokach N.A., Kuznetsov M.L., Gurzhiy V.V., Kukushkin V.Y. (2013). Selective Nucleophilic Oxygenation of Palladium-Bound Isocyanide Ligands: Route to Imine Complexes That Serve as Efficient Catalysts for Copper-/Phosphine-Free Sonogashira Reactions. Organometallics.

[35] Thomas S.P., Satheeshkumar K., Mugesh G., Guru Row T.N. (2015). Unusually Short Chalcogen Bonds Involving Organoselenium: Insights into the Se–N Bond Cleavage Mechanism of the Antioxidant Ebselen and Analogues. Chem. A Eur. J..

[36] Bortoli M., Ahmad S.M., Hamlin T.A., Bickelhaupt F.M., Orian L. (2018). Nature and strength of chalcogen–π bonds. Phys. Chem. Chem. Phys..

[37] Matsulevich Z.V., Lukiyanova J.M., Naumov V.I., Borisova G.N., Osmanov V.K., Borisov A.V., Grishina M.M., Khrustalev V.N. (2019). Bromination of bis(pyridin-2-yl) diselenide in methylene chloride: The reaction mechanism and crystal structures of 1H-pyridine-2-selenenyl dibromide and its cycloadduct with cyclopentene(3aSR,9aRS)-2,3,3a,9a-tetrahydro-1H-cyclopenta[4,5][1,3]selenazolo[. Acta Crystallogr. Sect. E Crystallogr. Commun..

[38] Khrustalev V.N., Matsulevich Z.V., Aysin R.R., Lukiyanova J.M., Fukin G.K., Zubavichus Y.V., Askerov R.K., Maharramov A.M., Borisov A.V. (2016). An unusually stable pyridine-2-selenenyl chloride: Structure and reactivity. Struct. Chem..

[39] Battye T.G.G., Kontogiannis L., Johnson O., Powell H.R., Leslie A.G.W. (2011). iMOSFLM: A new graphical interface for diffraction-image processing with MOSFLM. Acta Crystallogr. Sect. D Biol. Crystallogr..

[40] Evans P. (2006). Scaling and assessment of data quality. Acta Crystallogr. Sect. D Biol. Crystallogr..

[41] (2018). No Title.

[42] Krause L., Herbst-Irmer R., Sheldrick G.M., Stalke D. (2015). Comparison of silver and molybdenum microfocus X-ray sources for single-crystal structure determination. J. Appl. Crystallogr..

[43] Sheldrick G.M. (2015). SHELXT—Integrated space-group and crystal-structure determination. Acta Crystallogr. Sect. A Found. Crystallogr..

[44] Sheldrick G.M. (2015). Crystal structure refinement with SHELXL. Acta Crystallogr. Sect. C Struct. Chem..

[45] Lin Y.-S., Li G.-D., Mao S.-P., Chai J.-D. (2013). Long-Range Corrected Hybrid Density Functionals with Improved Dispersion Corrections. J. Chem. Theory Comput..

[46] Weigend F., Ahlrichs R. (2005). Balanced basis sets of split valence, triple zeta valence and quadruple zeta valence quality for H to Rn: Design and assessment of accuracy. Phys. Chem. Chem. Phys..

[47] Lu T., Chen F. (2012). Multiwfn: A multifunctional wavefunction analyzer. J. Comput. Chem..

[48] Glendening E.D., Badenhoop J.K., Reed A.E., Carpenter J.E., Bohmann J.A., Morales C.M., Weinhold F. (2004). NBO 5.G.

[49] Humphrey W., Dalke A., Schulten K. (1996). VMD: Visual molecular dynamics. J. Mol. Graph..

